# Brown Fat Dnmt3b Deficiency Ameliorates Obesity in Female Mice

**DOI:** 10.3390/life11121325

**Published:** 2021-11-30

**Authors:** Fenfen Li, Xin Cui, Jia Jing, Shirong Wang, Huidong Shi, Bingzhong Xue, Hang Shi

**Affiliations:** 1 Department of Biology, Georgia State University, Atlanta, GA 30303, USA; fli3@gsu.edu (F.L.); xcui@gsu.edu (X.C.); jjing@gsu.edu (J.J.); swang38@student.gsu.edu (S.W.); 2 Georgia Cancer Center, Medical College of Georgia, Augusta University, Augusta, GA 30912, USA; HSHI@augusta.edu

**Keywords:** Dnmt3b, brown adipocytes, thermogenesis, obesity

## Abstract

Obesity results from a chronic energy imbalance due to energy intake exceeding energy expenditure. Activation of brown fat thermogenesis has been shown to combat obesity. Epigenetic regulation, including DNA methylation, has emerged as a key regulator of brown fat thermogenic function. Here we aimed to study the role of Dnmt3b, a DNA methyltransferase involved in de novo DNA methylation, in the regulation of brown fat thermogenesis and obesity. We found that the specific deletion of Dnmt3b in brown fat promotes the thermogenic and mitochondrial program in brown fat, enhances energy expenditure, and decreases adiposity in female mice fed a regular chow diet. With a lean phenotype, the female knockout mice also exhibit increased insulin sensitivity. In addition, Dnmt3b deficiency in brown fat also prevents diet-induced obesity and insulin resistance in female mice. Interestingly, our RNA-seq analysis revealed an upregulation of the PI3K-Akt pathway in the brown fat of female Dnmt3b knockout mice. However, male Dnmt3b knockout mice have no change in their body weight, suggesting the existence of sexual dimorphism in the brown fat Dnmt3b knockout model. Our data demonstrate that Dnmt3b plays an important role in the regulation of brown fat function, energy metabolism and obesity in female mice.

## 1. Introduction

Obesity poses a serious risk for the development of a number of metabolic diseases such as type 2 diabetes, hypertension, dyslipidemia, and cardiovascular diseases [1]. Obesity results from a chronic energy excess due to energy intake exceeding energy expenditure [1]. Adaptive thermogenesis, in which brown fat thermogenesis is a major contributor, is an integral part of overall energy expenditure that also includes the basic metabolic rate and physical activity [2]. Brown fat thermogenesis traditionally was thought to rely on the mitochondrial inner membrane protein UCP1, which uncouples oxidative phosphorylation from ATP production, thereby profoundly increasing energy dissipation and overall energy expenditure [1,3,4,5]. Recent studies also revealed UCP1-independent thermogenesis mediated by SERCA2b-mediated calcium cycling or creatine-driven substrate cycling [6,7]. There exist two kinds of brown adipocytes in rodents: the classic brown adipose tissue (BAT) that is mainly located in the interscapular area and beige adipocytes that are scattered in white adipose tissue (WAT) and can be induced by β-adrenergic activation through β-adrenergic agonist treatment or cold exposure [8,9,10,11,12]. Activation of brown and beige adipocyte thermogenesis has been an efficient way to ameliorate obesity in rodents [13,14,15]. The recent rediscovery of functional brown fat in humans indicates that brown fat thermogenesis might be physiologically relevant to human energy metabolism [16,17,18].

Obesity is a complex disease that results from the interaction between genes and environmental factors (e.g., diets). Epigenetic regulation, a molecular link between obesity and environmental factors [19,20], has emerged as a key mechanism underlying the regulation of brown fat development and function. The DNA methylation of cytosine at CpG sites is a common epigenetic modification that frequently occurs in gene promoters and 5′ regions, where CpGs are often enriched [21,22]. DNA hypomethylation at the promoters normally activates gene transcription, while hypermethylation results in gene silencing [22]. Three DNA methyltransferases (DNMTs), namely DNMT1, -3a and -3b, are the main enzymes that catalyze the process of DNA methylation by adding methyl groups to CpG dinucleotides [22]. While DNMT1 is mainly involved in the maintenance of DNA methylation during DNA replication, Dnmt3a and -3b are responsible for de novo methylation [22]. We recently identified several epigenetic pathways including DNA methylation as a key regulator of adipocyte development and brown adipocyte thermogenesis [23,24,25,26,27]. For example, we discovered a biphasic role of DNA methylation, catalyzed by DNMT1 and -3a, in regulating adipogenesis, promoting differentiation at an early stage, while inhibiting lipogenesis at a late stage of 3T3-L1 differentiation [24]. In addition, we also found that the specific deletion of DNMT1 or -3a in brown adipocytes impairs cold-induced thermogenesis and promotes diet-induced obesity in mice [28]. In this study, we extended our interrogation into the role of Dnmt3b in the regulation of brown fat function. We have generated mice with a specific deletion of Dnmt3b in brown adipocytes. We then characterized the metabolic phenotypes and examined the thermogenic program in these mice fed a regular chow or high fat diet (HFD).

## 2. Materials and Methods

Mice. Mice with a brown fat-specific Dnmt3b knockout (UCP1-Cre::Dnmt3b-fl/fl, or D3bKO) were generated by crossing Dnmt3b-floxed mice, obtained from Mutant Mouse Regional Resource Centers (MMRRC, stock #029887), with Ucp1-Cre mice (Jackson Laboratory, Stock No 024570), where Cre is specifically expressed in brown fat and UCP1-positive beige adipocytes under the control of the Ucp1 promoter. The Dnmt3b-floxed mouse was created by inserting two loxP sites flanking exons 16–19, which encode the catalytic motif [29], and was backcrossed to a B6 background for more than five generations.

Metabolic measurement. The animal procedures conducted in this study were approved by the Institutional Animal Care and Use Committee at Georgia State University (GSU). All mice were housed in the GSU animal facility with a temperature- and humidity-controlled environment in a 12 h/12 h light–dark cycle and had access to water and food ad libitum. D3bKO mice and their flox/flox (fl/fl) littermate controls were fed either a chow diet or an HFD (Research Diets D12492, 60% of calories from fat) diet for up to 36 weeks. During the study, body weight was measured weekly. Food intake was measured in single-housed animals over seven consecutive days. The body composition of fat and lean mass was measured using a Minispec NMR body composition analyzer (Bruker BioSpin Corporation; Billerica, MA, USA). Energy expenditure, including oxygen consumption and locomotor activity, were measured using PhenoMaster metabolic cage systems (TSE Systems, Chesterfield, MO, USA). The data of energy expenditure were further analyzed by using the online software CalR at the website https://calrapp.org/ (accessed on 20 August 2021) as previously described [30]. Blood glucose was measured by a OneTouch Ultra Glucose meter (LifeScan, Milpitas, CA, USA). Insulin sensitivity was assessed by glucose tolerance and insulin tolerance tests (GTT and ITT, respectively) as we have previously described [31]. At the end of experiments, various tissues, including all fat depots and the liver were collected for the analysis of gene expression, protein content and immunohistochemistry.

Quantitative RT-PCR. Quantitative RT-PCR was conducted as we have previously described [31,32]. Briefly, total RNA from fat tissues was extracted using the Tri Reagent kit (Molecular Research Center, Cincinnati, OH, USA). The mRNA levels of gene expression were measured by a one-step quantitative RT-PCR with a TaqMan Universal PCR Master Mix kit (ThermoFisher Scientific, Waltham, MA, USA) using an Applied Biosystems QuantStudio 3 real-time PCR system (ThermoFisher Scientific) as we have previously described [31,32]. The sequences of the primer and probe pairs for the genes measured are as follows. UCP1: Forward 5′-CACCTTCCCGCTGGACACT-3′; Reverse 5′-CCCTAGGACACCTTTATACCTAATGG-3′; Probe 5′-AGCCTGGCCTTCACCTTGGATCTGA-3′. Cyclophilin: Forward 5′-GGTGGAGAGCACCAAGACAGA-3′; Reverse 5′-GCCGGAGTCGACAATGATG-3′; Probe 5′-ATCCTTCAGTGGCTTGTCCCGGCT-3′. Other TaqMan primers/probes were purchased from Applied Biosystems (ThermoFisher Scientific) as listed in Supplemental Table S1.

Immunoblotting. Immunoblotting measurement of the protein content was conducted as we described in [31]. Adipose tissues were homogenized in a modified radioimmunoprecipitation assay (RIPA) lysis buffer supplemented with a 1% protease inhibitor mixture and 1% phosphatase inhibitor mixture (Sigma-Aldrich, St. Louis, MO, USA), and homogenates were centrifuged. Tissue lysates were resolved by SDS-PAGE, which was transferred to nitrocellulose membranes (Bio-Rad, Hercules, CA, USA), followed by blocking, washing, incubating with primary/secondary antibodies, and developing with a Li-COR Imager System (Li-COR Biosciences, Lincoln, NE, USA). Primary and secondary antibodies used were as follows: anti-UCP1 antibody (1:500, abcam, ab23841); mitochondrial total OXPHOS protein antibody set (Abcam, ab110413); Alexa Fluor 680-conjugated secondary antibodies (Life Science Technologies); anti-GAPDH antibody (1:1000, Santa Cruz Biotechnology, sc-32233); anti-α-Tubulin antibody (1:1000, Advanced BioChemicals, ABCENT4777).

Immunohistochemistry (IHC). Fat tissues were fixed in 10% neutral formalin, embedded in paraffin and sectioned at a 5 µm thickness. The sections were further processed for hematoxylin and eosin (H&E) staining or immunostaining with a UCP1 antibody (1:150, abcam, ab10983) as we have previously described [32,33,34].

RNA-sequencing analysis. Total RNA was extracted from adipose tissue as described above and was commercially sequenced by Beijing Genomics Institute (BGI, Shenzhen, China). Bioinformatics analysis was conducted as we have previously described [32]. Briefly, after the removal of raw reads adaptor sequences and low-quality data via a filter, clean reads were mapped to reference sequences (University of California Santa Cruz Mouse Genome Browser mm9 Assembly) using SOAPaligner/SOAP2. Reads per kilobase per million reads (RPKM) were calculated to represent the gene expression level, and were used for comparing differentially expressed genes (DEGs) between the two genotypes fl/fl and D3bKO, with thresholds set at a more than two-fold increase or more than 50% decrease, and a false discovery rate (FDR) of <0.001. DEGs were further used for Gene Ontology (GO) and pathway enrichment analysis.

Statistics. Data were expressed as mean ± SEM. Groups were compared for difference analysis by a one-way ANOVA or *t*-test as appropriate. Statistical significance is considered at *p* < 0.05.

## 3. Results

### 3.1. Dnmt3b Deficiency in Brown Fat Decreases Adiposity in Female Mice Fed a Chow Diet

**Figure 1:**
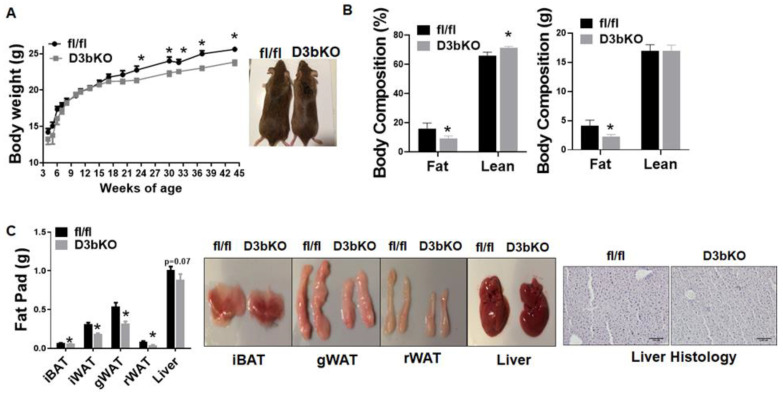
Dnmt3b deficiency in brown fat reduces adiposity in female mice fed a chow diet. Five-week-old female D3bKO and littermate control fl/fl mice were put on a regular chow diet for 40 weeks. (**A**) Body weight growth curve in female D3bKO and fl/fl mice. (**B**) Body composition measured by a Bruker NMR body composition analyzer in female D3bKO and fl/fl mice. (**C**) Organ weight of interscapular brown adipose tissue (iBAT), inguinal white adipose tissue (iWAT), gonadal WAT (gWAT), retroperitoneal WAT (rWAT), and liver in female D3bKO and fl/fl mice. H&E staining of liver in female D3bKO and fl/fl mice (left panel). All data are expressed as mean ± SEM; *n* = 7–10/group; * *p* < 0.05 vs. fl/fl.

**Figure 2:**
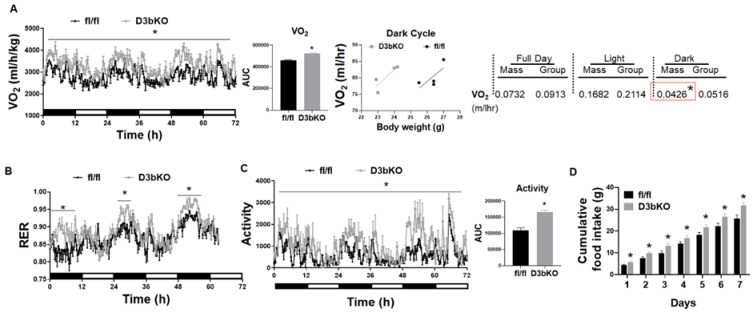
Dnmt3b deficiency in brown fat promotes energy expenditure in female mice fed a chow diet. Thirty-six week-old female D3bKO and fl/fl mice fed a chow diet were put in a TSE PhenoMaster metabolic cage system for metabolic characterization. (**A**) Oxygen consumption. The data of oxygen consumption was further analyzed by a regression-based analysis of covariance (ANCOVA) using the online software CalR at the website https://calrapp.org/ (accessed on 20 August 2021). The regression analysis of absolute VO2/hr against body weight was plotted (middle panel) and revealed a body weight-independent effect in the night cycle (*p* = 0.0426 right panel). (**B**) Respiratory exchange ratio (RER). (**C**) Locomotor activity. (**D**) Cumulative food intake. All data are expressed as mean ± SEM; *n* = 4/group; * *p* < 0.05 vs. fl/fl.

**Figure 3:**
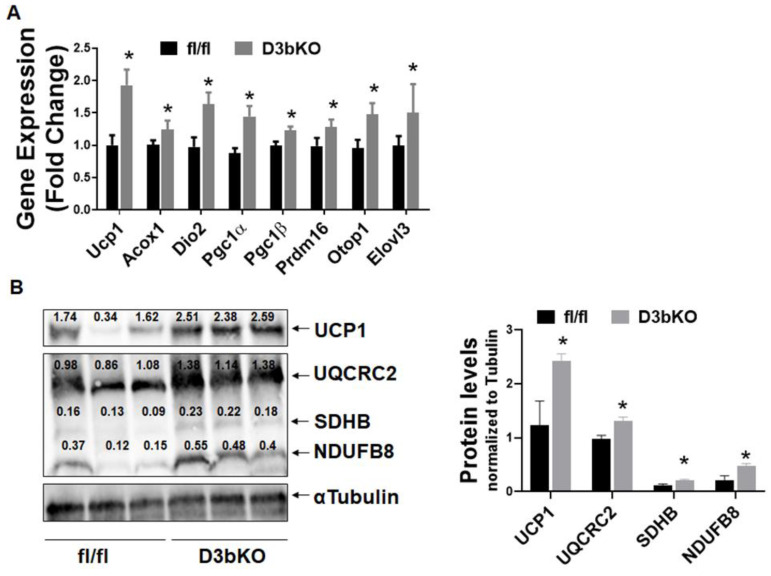
Dnmt3b deficiency promotes the brown fat thermogenic program in female mice. Five-week-old female D3bKO and littermate control fl/fl mice were put on a regular chow diet for 40 weeks. (**A**) Quantitative RT-PCR analysis of thermogenic gene expression in iBAT (*n* = 7/group). (**B**) Immunoblotting of UCP1 and mitochondrial respiratory chain complex proteins in iBAT (*n* = 3/group). The number on each band represents the ratio of the densitometry of the band, normalized by its corresponding control tubulin. All data are expressed as mean ± SEM; * *p* < 0.05 vs. fl/fl.

To determine the role of brown fat Dnmt3b in the regulation of energy metabolism and body weight, we generated mice with a specific deletion of Dnmt3b in brown fat (D3bKO) by crossing Dnmt3b-floxed mice with Ucp1-cre mice. The Dnmt3b deletion in brown fat resulted in an almost 50% reduction of Dnmt3b mRNA expression without changes of Dnmt1 and Dnmt3a expression (Supplementary Figure S1). We first characterized the metabolic phenotype of female D3bKO mice fed a chow diet. Figure 1A shows that the body weight between D3bKO mice and their fl/fl littermate controls started diverging after they were 18 weeks old and the difference became more evident over the course of aging, in which a lower weight was observed in female D3bKO mice. Using an NMR body composition analyzer, we found a significant decrease in the fat composition while there was a reciprocal increase in the lean composition in 10-month-old D3bKO mice compared to their littermate controls (Figure 1B). It was consistent that these aged female D3bKO mice also exhibited a decreased fat mass in various fat depots, including interscapular BAT (iBAT), inguinal WAT (iWAT), gonadal WAT (gWAT), and retroperitoneal WAT (rWAT) (Figure 1C). We also observed a tendency towards a decreased liver weight and hepatic lipid accumulation in D3bKO mice (Figure 1C). Using a PhenoMaster metabolic cage system, we found that female D3bKO mice displayed a higher oxygen consumption (VO2) (Figure 2A, left panel). The data of oxygen consumption was further analyzed by a regression-based analysis of covariance (ANCOVA) [35] using the online software CalR at the website https://calrapp.org/ (accessed on 20 August 2021) as previously described [30]. The regression analysis of absolute VO2/hr against body weight revealed a body weight-independent effect on oxygen consumption during the dark cycle (*p* = 0.0426, Figure 2A middle and right panel), indicating that brown fat Dnmt3b deficiency increases oxygen consumption in the dark cycle when mice are most active in food intake and other activities. Moreover, female D3bKO mice also had an increase in their respiratory exchange ratio (RER) (Figure 2B), indicating that these mice preferentially used glucose as an energy fuel. In addition, increased locomotor activity was observed in female D3bKO mice (Figure 2C), which may contribute to the enhanced overall energy expenditure. Surprisingly, the knockout mice had an increased food intake (Figure 2D), which may compensate for the increased energy expenditure. Further quantitative RT-PCR analysis revealed increased expression of a panel of thermogenic genes, such as Ucp1, Dio2, Pgc1α, Pgc1β, Prdm16, Otop1, etc., (Figure 3A) in the iBAT of female D3bKO mice, which was associated with increased UCP1 protein levels measured by immunoblotting (Figure 3B). We further examined mitochondrial respiratory chain proteins by immunoblotting, which showed an upregulation of the succinate dehydrogenase complex subunit B (SDHB) and the complex III cytochrome b-c1 complex subunit 2 (CIII-UQCRC2) in the iBAT of D3bKO mice (Figure 3B). These data suggest that an enhanced thermogenic and mitochondrial program may contribute to the increased energy expenditure and decreased adiposity in female D3bKO mice.

**Figure 4:**
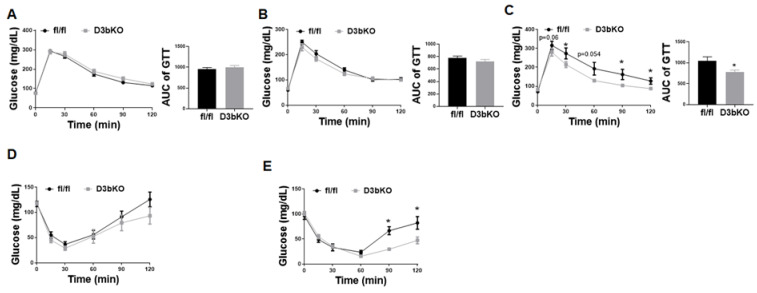
Dnmt3b deficiency in brown fat improves insulin sensitivity in aged female mice. Five-week-old female D3bKO and littermate control fl/fl mice were put on a regular chow diet for 40 weeks. (**A**) GTT in 17-week-old female D3bKO and fl/fl mice. (**B**) GTT in 20-week-old female D3bKO and fl/fl mice. (**C**) GTT in 36-week-old female D3bKO and fl/fl mice. (**D**) ITT in 21-week-old female D3bKO and fl/fl mice. (**E**) ITT in 37-week-old female D3bKO and fl/fl mice All data are expressed as mean ± SEM; *n* = 7–10/group; * *p* < 0.05 vs. fl/fl.

Since glucose homeostasis and insulin sensitivity are closely associated with adiposity levels, we therefore characterized these metabolic parameters with GTTs and ITTs in female D3bKO mice. Interestingly, at the beginning of the body weight divergence when the difference was not eminent, there was no change in GTTs between the knockout mice and their controls at the age of 17 weeks (Figure 4A). The trend moved towards more glucose tolerance for the female knockout mice at the age of 20 weeks (Figure 4B), the difference of which eventually became evident at the age of 36 weeks when the body weight of female D3bKO mice was significantly lower (Figure 4C). Similar results were observed in ITTs. There was a tendency towards increased insulin sensitivity, albeit not significant, for D3bKO mice at the age of 21 weeks (Figure 4D). Eventually female D3bKO mice became more insulin sensitive at the age of 36 weeks (Figure 4E). These data suggest that with a lesser tendency to develop obesity during the aging process, female D3bKO mice are protected from age-related insulin resistance.

**Figure 5:**
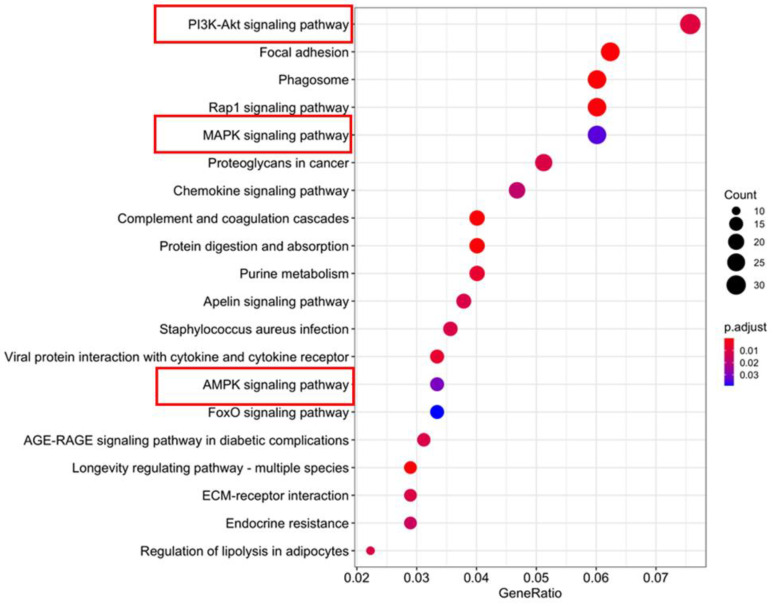
RNA-seq analysis reveals an upregulation of the PI3-Akt signaling pathway in the iBAT of female D3bKO and fl/fl mice fed a chow diet. Five-week-old female D3bKO and littermate control fl/fl mice were put on a regular chow diet for 40 weeks. RNA-seq analysis was conducted using the iBAT of 40-week-old female D3bKO and fl/fl mice.

We performed an RNA-seq analysis using brown fat from the female D3bKO mice and the littermate control fl/fl mice to unbiasedly examine the gene expression profiles. There were 928 genes differentially regulated by Dnmt3b deficiency, among which 513 genes were upregulated and 415 genes were downregulated in the Dnmt3b-deficient iBAT. Interestingly, KEGG gene enrichment analysis disclosed a number of metabolic pathways, among which PI3-Akt and MAPK, two downstream pathways of insulin signaling, were top-ranked (Figure 5). Upregulation of PI3-Akt, MAPK and AMPK pathways by Dnmt3b deficiency may enhance insulin sensitivity and facilitate glucose metabolism, as manifested in GTT and ITT in D3bKO mice.

### 3.2. Dnmt3b Deficiency in Brown Fat Ameliorates Diet-Induced Obesity in Female Mice

**Figure 6:**
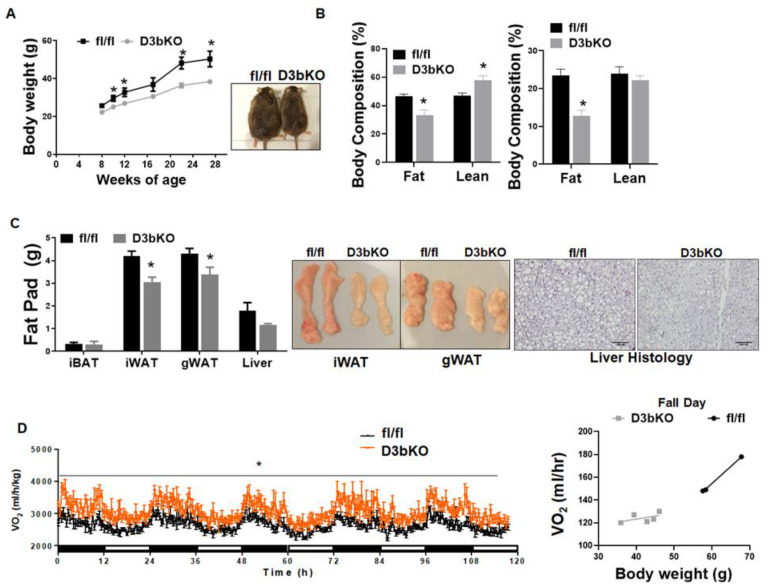
Dnmt3b deficiency in brown fat prevents HFD-induced obesity in female mice. Eight-week-old female D3bKO and littermate control fl/fl mice were put on an HFD for 20 weeks. (**A**) Body weight growth curve in female D3bKO and fl/fl mice. (**B**) Body composition measured by a Bruker NMR body composition analyzer in female D3bKO and fl/fl mice. (**C**) Organ weight of interscapular brown adipose tissue (iBAT), inguinal white adipose tissue (iWAT), gonadal WAT (gWAT), and liver in the female D3bKO and fl/fl mice fed HFD. H&E staining of liver in female D3bKO and fl/fl mice (right panel). (**D**) Oxygen consumption measured by a TSE PhenoMaster metabolic cage system. The data of oxygen consumption were further analyzed by regression-based analysis of covariance (ANCOVA) using the online software CalR at the website https://calrapp.org/ (accessed on 20 August 2021). The regression analysis of absolute VO2/hr against body weight was plotted (right panel) and revealed a body weight-dependent effect (ANCOVA *p* = 0.45). All data are expressed as mean ± SEM; *n* = 4–6/group; * *p* < 0.05 vs. fl/fl.

**Figure 7:**
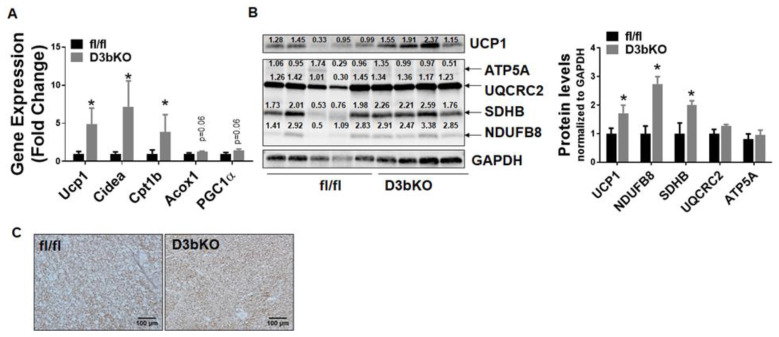
Dnmt3b deficiency promotes the brown fat thermogenic program in female mice fed HFD. Eight-week-old female D3bKO and littermate control fl/fl mice were put on an HFD for 20 weeks. (**A**) Quantitative RT-PCR analysis of thermogenic gene expression in iBAT (*n* = 4–6/group). (**B**) Immunoblotting of UCP1 and mitochondrial respiratory chain complex proteins in iBAT (*n* = 4–5/group). The number on each band represents the ratio of the densitometry of the band, normalized by its corresponding control GAPDH. (**C**) Immunohistochemical (IHC) staining of UCP1 in iBAT. All data are expressed as mean ± SEM; * *p* < 0.05 vs. fl/fl.

**Figure 8:**
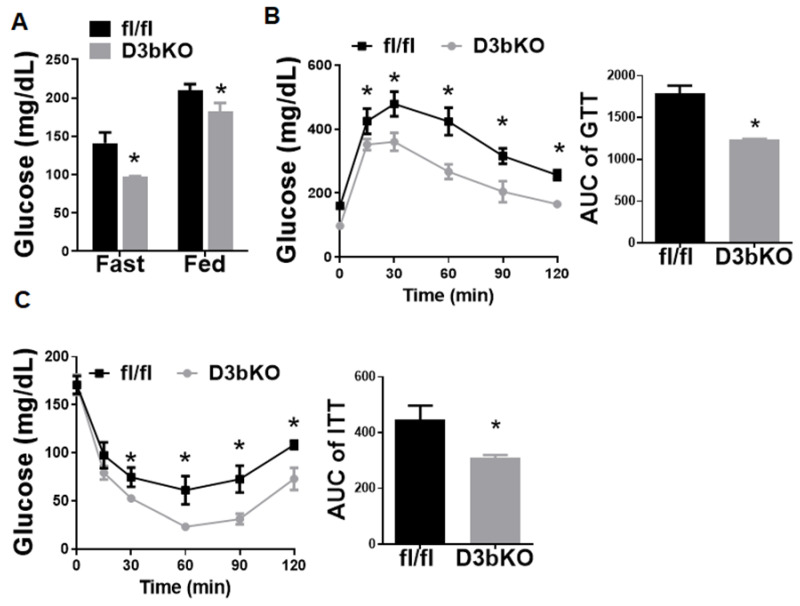
Dnmt3b deficiency in brown fat improves insulin sensitivity in female mice fed HFD. Eight-week-old female D3bKO and littermate control fl/fl mice were put on an HFD for 20 weeks. (**A**) Fasting and fed glucose levels. (**B**) GTT in 24-week-old female D3bKO and fl/fl mice. (**C**) ITT in 26-week-old female D3bKO and fl/fl mice. All data are expressed as mean ± SEM; *n* = 4–6/group; * *p* < 0.05 vs. fl/fl.

To determine the role of brown fat Dnmt3b in the regulation of diet-induced obesity, we further conducted a metabolic characterization of body weight, energy metabolism and insulin sensitivity in female D3bKO mice fed HFD. As shown in Figure 6A, female D3bKO mice gained significantly less weight compared to the control fl/fl mice. The NMR body composition analysis revealed a significant decrease in the body fat composition while there was a corresponding increase in the lean composition in female D3bKO mice (Figure 6B). It was consistent that female D3bKO mice had a lower fat mass in two major fat depots iWAT and gWAT, with a tendency towards a lower liver weight (Figure 6C). Our H&E staining also revealed a tendency of decreased hepatic lipid accumulation in D3bKO mice (Figure 6C right panel). Using a PhenoMaster metabolic cage system, we found that D3bKO mice exhibited enhanced oxygen consumption, normalized to body weight (Figure 6D left panel). A further regression analysis of absolute VO2/hr against body weight revealed a body weight-dependent effect on oxygen consumption during the full day cycle (Figure 6D right panel). Due to the unchanged RER, locomotor activity and food intake (Supplementary Figure S2), it is still likely that an increased energy expenditure may account for the lean phenotype observed in D3bKO mice fed HFD. In support of the increased energy expenditure, quantitative PCR analysis revealed an increase in the expression of thermogenic genes, including Ucp1, Cidea and Cpt1b (Figure 7A). This was consistent with increased UCP1 protein and mitochondrial respiratory chain proteins, NADH dehydrogenase 1β subcomplex 8 (NDUFB8) and succinate dehydrogenase complex subunit B (SDHB), measured by immunoblotting (Figure 7B). The immunohistochemical analysis further confirmed more UCP1 staining and smaller brown adipocytes in the iBAT of D3bKO mice (Figure 7C). We further characterized the glucose tolerance and insulin sensitivity with GTTs and ITTs in female D3bKO mice fed HFD. With decreased adiposity, the knockout mice had lower fasting and fed blood glucose levels (Figure 8A). It was consistent that female D3bKO mice were more glucose tolerant and insulin sensitive than their fl/fl controls, as assessed by GTT and ITT, respectively (Figure 8B,C).

Unlike the lean phenotype observed in female D3bKO mice, there was no change in the body weight of male D3bKO mice fed a regular chow diet (Supplementary Figure S3A). Moreover, there was no difference in the body composition and fat pad weight between male D3bKO mice and their controls (Supplementary Figure S3B,C). This was consistent with no changes in oxygen consumption, RER, locomotor activity and food intake between male D3bKO mice and their littermate controls (Supplementary Figure S4A–D). Immunoblotting analysis showed decreased UCP1 protein in the iBAT of male D3bKO mice (Supplementary Figure S5). Male D3bKO mice had increased levels of fed blood glucose (Supplementary Figure S6A) and exhibited glucose intolerance and insulin resistance assessed by GTT and ITT, respectively (Supplementary Figure S6B,C).

## 4. Discussion

In the present study, we discovered that the specific deletion of Dnmt3b in brown fat promotes the thermogenic program in brown fat, enhances energy expenditure, decreases adiposity and improves insulin sensitivity in female mice fed a regular chow diet or HFD. The plausibility of this study was derived from a growing body of evidence suggesting a role of epigenetic regulation in obesity and metabolic disorders. Obesity is a complex disease that results from gene and environment interactions. Epigenetic regulation has emerged as a molecular link between environmental factors (e.g., diets) and obesity [36,37,38,39,40]. Alterations of DNA methylation, a common epigenetic modification, have also been identified in genes involved in various metabolic pathways, such as Pparγ [41], Pgc1α [42,43], leptin [36,44], Lpl and aP2 [41], etc. We have also reported that epigenetic regulation by DNA methylation plays a critical role in modulating various metabolic pathways, including neuronal control of energy metabolism in obesity, macrophage polarization and inflammation in type 2 diabetes and atherosclerosis, and adipocyte differentiation [24,25,31,45,46,47]. Since brown fat thermogenesis is an integral part of overall energy homeostasis, we recently extended our interest towards the understanding of DNA methylation in brown fat development and thermogenic function [28,32]. We discovered that the specific deletion of Dnmt1 or -3a in brown fat causes brown fat remodeling by converting functional brown fat into skeletal myocyte-like brown fat, resulting in decreased energy expenditure and increased adiposity [28]. It is consistent that the genetic model with a Dnmt3b deletion in brown fat-skeletal lineage cells largely recapitulates the phenotypes of the models with a brown fat Dnmt1 or -3a knockout [32]. It should be noted that the Cre lines used to generate these models express the Cre recombinase at different developmental stages. The model with a Dnmt3b deficiency in brown fat-skeletal muscle lineage cells was generated by the Myf5-Cre line that deletes Dnmt3b at the early developmental stage of progenitor cells, while the model with a Dnmt1 or -3a deficiency in brown fat was achieved by the Ucp1-Cre line that deletes the floxed genes in relatively mature brown adipocytes. By contrast, here we found that female D3bKO mice with a Dnmt3b deletion in brown fat show an opposite phenotype with increased energy expenditure and decreased adiposity. Although the exact mechanism underlying the discrepancy is not clear, it is conceivable that Dnmt3b may play different roles at different stages of brown fat development. On the other hand, even in mature brown adipocytes Dnmt1/3a and Dnmt3b may have different targets in regulating biological pathways. We have shown that DNMT1 or -3a serves to maintain brown adipocyte identity by repressing myogenic remodeling via MyoD1 methylation in mature brown adipocytes, thus promoting active brown adipocyte thermogenic function [28]. However, the opposite phenotype observed in D3bKO mice likely suggests a different mechanism that may involve repression of the thermogenic program instead of the myogenic program by Dnmt3b. Indeed, the promoter regions of several key thermogenic genes, such as Prdm16, Pgc1α and Ucp1, are enriched with CpG sites that are subject to modifications by DNA methylation [48,49,50], and their demethylation by Dnmt3b deficiency may promote the expression of these genes. Indeed, our quantitative PCR analysis revealed the increased expression of Prdm16, Pgc1α and Ucp1 in Dnmt3b-deficient brown fat. Taken together, DNA methylation catalyzed by Dnmt1, -3a and -3b has a complex role in the regulation of brown fat development and thermogenic function depending on the developmental stages and pathway-specific functions of the individual methyltransferase. Future studies are warranted to determine the signaling pathway(s) mediating the inhibitory effect of brown fat Dnmt3b on thermogenic function.

A regression analysis of the absolute VO2/hr against body weight revealed a body weight-dependent effect on oxygen consumption in female D3bKO mice fed HFD (ANCOVA *p* = 0.453) (Figure 6D right panel), which makes the case of increased energy expenditure less compelling. That being said, we still believe that the increased energy expenditure is still a likely factor responsible for the reduced adiposity in female D3bKO mice. For one, in both the chow and high fat diet fed mice, Dnmt3b deficiency increased the thermogenic program and mitochondrial respiratory protein levels in brown fat (Figure 3 and Figure 7), which likely increases overall energy expenditure. On the other hand, we did not observe any changes of food intake between the female D3bKO mice and their littermate controls, suggesting that the alteration of energy expenditure is still a likely factor responsible for the body weight difference. In the cohort of HFD-fed mice, a huge difference in body weight between the two genotype groups may obscure the change of energy expenditure, since we indeed found a body weight-independent effect on oxygen consumption in chow fed mice with a lesser difference in body weight. Future experiments that determine the metabolic rate prior to the weight change are warranted to discern the real contribution of increased energy expenditure to decreased adiposity in D3bKO mice.

In addition, there are still unaddressed questions regarding the metabolic phenotype of female D3bKO mice. These mice are hyperphagic while still remaining lean. The increased food intake may result from a secondary effect compensating for an increased energy expenditure. However, future studies are still required to pinpoint what factor(s) (e.g., brown fat secretory factors “batokines” or afferent sensory nerve activation) mediates the increased food intake. Moreover, the female D3bKO mice on the chow diet have increased RER, indicating that they preferentially utilize glucose as a fuel. This may contribute to the improved insulin sensitivity and glucose tolerance in these mice. Our RNA-seq analysis disclosed an upregulation of the PI3K-Akt pathway in Dnmt3b-deficient brown fat, which may contribute to the increased insulin sensitivity and glucose utilization in D3bKO mice besides their decreased adiposity. However, it is not clear what pathways in BAT, which are altered by the Dnmt3b deficiency, might be responsible for the increased PI3K-Akt signaling at the expression level. On the other hand, the improved insulin sensitivity in D3bKO mice may secondarily stem from a reduced body weight. As shown in Figure 4, glucose tolerance and insulin sensitivity assessed by GTTs and ITTs, respectively, showed no difference between the two genotype groups when body weight remained unchanged before the age of 20 weeks. It was not until the age of 36 weeks when the body weight of D3bKO mice trended lower, that the female D3bKO mice became more insulin sensitive.

Unlike the female D3bKO mice, male D3bKO mice did not show a change in body weight, but had an increased body weight gain, glucose intolerance and insulin resistance, an overall phenotype somewhat opposite to that of the female D3bKO mice. Sexual dimorphism of metabolic phenotypes is a common phenomenon in both humans and rodents. Females have a higher fat composition than males in humans [51]. Females and males have different fat distributions with fat storage in subcutaneous areas for females and visceral regions for males [52]. They also exhibit differential lipid metabolism, with a higher lipolytic rate and triglyceride secretion/clearance rate observed in females [53]. It has been well documented that estrogen and its receptors play a pivotal role in various metabolic and developmental processes, including metabolism, glucose homeostasis, differentiation, cell proliferation, immune and anti-inflammatory functions [54]. Interestingly, we recently reported that the promoter and 5′ region of the estrogen receptor (ERα), are enriched with CpG sites, which are subject to DNA methylation by DNMTs [45]. It is conceivable that estrogen may have an additional protective effect via increased receptor signaling due to the Dnmt3b deficiency in the female D3bKO mice, leading to the lean and insulin sensitive phenotype.

In summary, our data indicate that a Dnmt3b deficiency in brown fat promotes the thermogenic program, leading to enhanced energy expenditure and decreased adiposity in female mice. With a reduced body weight and fat mass, female D3bKO mice exhibit ameliorated metabolic phenotypes in age-related or diet-induced insulin resistance. However, male D3bKO mice have no change in body weight but exhibit insulin resistance, suggesting a sexual dimorphism of metabolism in the brown fat Dnmt3b knockout model. Our data demonstrate that Dnmt3b plays an important role in the regulation of brown fat thermogenic function, energy metabolism and obesity in female mice.

## Data Availability

All datasets will be available upon request to the corresponding authors Drs. Hang Shi and Bingzhong Xue.

## Supplementary Materials

The following are available online at https://www.mdpi.com/article/10.3390/life11121325/s1, Figure S1: Dnmt3b, Dnmt3a and Dnmt1 mRNA levels in the interscapular BAT (iBAT) of female D3bKO and fl/fl mice (*n* = 4/group). All data are expressed as mean ± SEM; * *p* < 0.05 vs. fl/fl. Figure S2: Metabolic characterization of female D3bKO and fl/fl control mice on HFD. 8-week old female D3bKO and their littermate control fl/fl mice were put on HFD for 20 weeks. Respiratory exchange ratio (RER) (A), locomotor activity (B), and food intake (C) were measured by TSE PhenoMaster metabolic cage systems in female D3bKO and fl/fl mice fed HFD. All data are expressed as mean ± SEM; *n* = 4/group; * *p* < 0.05 vs. fl/fl. Figure S3: Dnmt3b deficiency in brown fat does not change body weight in male mice. 5-week old male D3bKO and their littermate control fl/fl mice were put on a regular chow diet for 25 weeks. (A) Body weight growth curve in male D3bKO and fl/fl mice. (B) Body composition measured by a Bruker NMR body composition analyzer in male D3bKO and fl/fl mice. (C) Organ weight of interscapular brown adipose tissue (iBAT), inguinal white adipose tissue (iWAT), gonadal WAT (gWAT), retroperitoneal WAT (rWAT), and liver in female D3bKO and fl/fl mice. All data are expressed as mean ± SEM; *n* = 8–11/group; * *p* < 0.05 vs. fl/fl. Figure S4: Dnmt3b deficiency in brown fat does not change energy expenditure in male mice. 29-week old male D3bKO and fl/fl mice fed chow diet were put in TSE PhenoMaster metabolic cage system for the metabolic characterization. (A) Oxygen consumption. (B) Respiratory exchange ratio (RER). (C) Locomotor activity. (D) Cumulative food intake. All data are expressed as mean ± SEM; *n* = 4/group. Figure S5: Dnmt3b deficiency reduces UCP1 protein levels in the iBAT of male mice. 5-week old male D3bKO and their littermate control fl/fl mice were put on a regular chow diet for 25 weeks. All data are expressed as mean ± SEM; *n* = 4/group; * *p* < 0.05 vs. fl/fl. Figure S6: Dnmt3b deficiency in brown fat causes insulin resistance in male mice. 5-week old male D3bKO and their littermate control fl/fl mice were put on a regular chow diet for 25 weeks. (A) Fasting and fed glucose levels. (B) Glucose tolerance test (GTT) in 26-week male D3bKO and fl/fl mice. (C) Insulin tolerance test (ITT) in 28-week old male D3bKO and fl/fl mice. All data are expressed as mean ± SEM; *n* = 8–11/group. Table S1: TaqMan primers/probes from Applied Biosystems.

## References

[1] Hill J.O., Wyatt H.R., Peters J.C. (2012). Energy balance and obesity. Circulation.

[2] Lowell B.B., Spiegelman B.M. (2000). Towards a molecular understanding of adaptive thermogenesis. Nat. Cell Biol..

[3] Donahoo W., Levine A.J., Melanson E.L. (2004). Variability in energy expenditure and its components. Curr. Opin. Clin. Nutr. Metab. Care.

[4] Cannon B., Nedergaard J. (1985). The biochemistry of an inefficient tissue: Brown adipose tissue. Essays Biochem..

[5] Nicholls D.G., Locke R.M. (1984). Thermogenic mechanisms in brown fat. Physiol. Rev..

[6] Kazak L., Chouchani E.T., Jedrychowski M.P., Erickson B., Shinoda K., Cohen P., Vetrivelan R., Lu G.Z., Laznik-Bogoslavski D., Hasenfuss S.C. (2015). A Creatine-Driven Substrate Cycle Enhances Energy Expenditure and Thermogenesis in Beige Fat. Cell.

[7] Ikeda K., Kang Q., Yoneshiro T., Camporez J.P., Maki H., Homma M., Shinoda K., Chen Y., Lu X., Maretich P. (2017). UCP1-independent signaling involving SERCA2b-mediated calcium cycling regulates beige fat thermogenesis and systemic glucose homeostasis. Nat. Med..

[8] Ishibashi J., Seale P. (2010). Medicine. Beige can be slimming. Science.

[9] Petrovic N., Walden T.B., Shabalina I.G., Timmons J.A., Cannon B., Nedergaard J. (2010). Chronic peroxisome proliferator-activated receptor gamma (PPARgamma) activation of epididymally derived white adipocyte cultures reveals a population of thermogenically competent, UCP1-containing adipocytes molecularly distinct from classic brown adipocytes. J. Biol. Chem..

[10] Wu J., Bostrom P., Sparks L.M., Ye L., Choi J.H., Giang A.H., Khandekar M., Virtanen K.A., Nuutila P., Schaart G. (2012). Beige adipocytes are a distinct type of thermogenic fat cell in mouse and human. Cell.

[11] Brito M.N., Brito N.A., Baro D.J., Song C.K., Bartness T.J. (2007). Differential Activation of the Sympathetic Innervation of Adipose Tissues by Melanocortin Receptor Stimulation. Endocrinology.

[12] Brito N.A., Brito M.N., Bartness T.J. (2008). Differential sympathetic drive to adipose tissues after food deprivation, cold exposure or glucoprivation. Am. J. Physiol. Regul. Integr. Comp. Physiol..

[13] Cohen P., Levy J.D., Zhang Y., Frontini A., Kolodin D.P., Svensson K.J., Lo J.C., Zeng X., Ye L., Khandekar M.J. (2014). Ablation of PRDM16 and beige adipose causes metabolic dysfunction and a subcutaneous to visceral fat switch. Cell.

[14] Feldmann H.M., Golozoubova V., Cannon B., Nedergaard J. (2009). UCP1 ablation induces obesity and abolishes diet-induced thermogenesis in mice exempt from thermal stress by living at thermoneutrality. Cell Metab..

[15] Seale P., Conroe H.M., Estall J., Kajimura S., Frontini A., Ishibashi J., Cohen P., Cinti S., Spiegelman B.M. (2011). Prdm16 determines the thermogenic program of subcutaneous white adipose tissue in mice. J. Clin. Investig..

[16] Cypess A.M., Lehman S., Williams G., Tal I., Rodman D., Goldfine A.B., Kuo F.C., Palmer E.L., Tseng Y.H., Doria A. (2009). Identification and importance of brown adipose tissue in adult humans. N. Engl. J. Med..

[17] van Marken Lichtenbelt W.D., Vanhommerig J.W., Smulders N.M., Drossaerts J.M., Kemerink G.J., Bouvy N.D., Schrauwen P., Teule G.J. (2009). Cold-activated brown adipose tissue in healthy men. N. Engl. J. Med..

[18] Virtanen K.A., Lidell M.E., Orava J., Heglind M., Westergren R., Niemi T., Taittonen M., Laine J., Savisto N.J., Enerback S. (2009). Functional brown adipose tissue in healthy adults. N. Engl. J. Med..

[19] Edwards T.M., Myers J.P. (2007). Environmental exposures and gene regulation in disease etiology. Environ. Health Perspect..

[20] Skinner M.K., Manikkam M., Guerrero-Bosagna C. (2010). Epigenetic transgenerational actions of environmental factors in disease etiology. Trends Endocrinol. Metab..

[21] Luczak M.W., Jagodzinski P.P. (2006). The role of DNA methylation in cancer development. Folia Histochem. Cytobiol..

[22] Suzuki M.M., Bird A. (2008). DNA methylation landscapes: Provocative insights from epigenomics. Nat. Rev. Genet..

[23] Jing J., Li F., Zha L., Yang X., Wu R., Wang S., Xue B., Shi H. (2020). The histone methyltransferase Suv39h regulates 3T3-L1 adipogenesis. Adipocyte.

[24] Yang X., Wu R., Shan W., Yu L., Xue B., Shi H. (2016). DNA Methylation Biphasically Regulates 3T3-L1 Preadipocyte Differentiation. Mol. Endocrinol..

[25] Chen Y.S., Wu R., Yang X., Kou S., MacDougald O.A., Yu L., Shi H., Xue B. (2016). Inhibiting DNA methylation switches adipogenesis to osteoblastogenesis by activating Wnt10a. Sci. Rep..

[26] Li F., Wu R., Cui X., Zha L., Yu L., Shi H., Xue B. (2016). Histone Deacetylase 1 (HDAC1) Negatively Regulates Thermogenic Program in Brown Adipocytes via Coordinated Regulation of Histone H3 Lysine 27 (H3K27) Deacetylation and Methylation. J. Biol. Chem..

[27] Zha L., Li F., Wu R., Artinian L., Rehder V., Yu L., Liang H., Xue B., Shi H. (2015). The Histone Demethylase UTX Promotes Brown Adipocyte Thermogenic Program Via Coordinated Regulation of H3K27 Demethylation and Acetylation. J. Biol. Chem..

[28] Li F., Jing J., Movahed M., Cui X., Cao Q., Wu R., Chen Z., Yu L., Pan Y., Shi H. (2020). Epigenetic Interaction between UTX and DNMT1 Regulates Diet-Induced Myogenic Remodeling in Brown Fat. bioRxiv.

[29] Dodge J.E., Okano M., Dick F., Tsujimoto N., Chen T., Wang S., Ueda Y., Dyson N., Li E. (2005). Inactivation of Dnmt3b in mouse embryonic fibroblasts results in DNA hypomethylation, chromosomal instability, and spontaneous immortalization. J. Biol. Chem..

[30] Mina A.I., LeClair R.A., LeClair K.B., Cohen D.E., Lantier L., Banks A.S. (2018). CalR: A Web-Based Analysis Tool for Indirect Calorimetry Experiments. Cell Metab..

[31] Wang X., Cao Q., Yu L., Shi H., Xue B., Shi H. (2016). Epigenetic regulation of macrophage polarization and inflammation by DNA methylation in obesity. JCI Insight.

[32] Wang S., Cao Q., Cui X., Jing J., Li F., Shi H., Xue B., Shi H. (2021). Dnmt3b Deficiency in Myf5+-Brown Fat Precursor Cells Promotes Obesity in Female Mice. Biomolecules.

[33] Nguyen N.L., Barr C.L., Ryu V., Cao Q., Xue B., Bartness T.J. (2017). Separate and shared sympathetic outflow to white and brown fat coordinately regulates thermoregulation and beige adipocyte recruitment. Am. J. Physiol. Regul. Integr. Comp. Physiol..

[34] Cao Q., Jing J., Cui X., Shi H., Xue B. (2019). Sympathetic nerve innervation is required for beigeing in white fat. Physiol. Rep..

[35] Muller T.D., Klingenspor M., Tschop M.H. (2021). Revisiting energy expenditure: How to correct mouse metabolic rate for body mass. Nat. Metab..

[36] Campion J., Milagro F.I., Martinez J.A. (2009). Individuality and epigenetics in obesity. Obes. Rev..

[37] Holness M.J., Sugden M.C. (2006). Epigenetic regulation of metabolism in children born small for gestational age. Curr. Opin. Clin. Nutr. Metab. Care.

[38] Ling C., Groop L. (2009). Epigenetics: A molecular link between environmental factors and type 2 diabetes. Diabetes.

[39] Maier S., Olek A. (2002). Diabetes: A candidate disease for efficient DNA methylation profiling. J. Nutr..

[40] Szarc vel Szic K., Ndlovu M.N., Haegeman G., Vanden Berghe W. (2010). Nature or nurture: Let food be your epigenetic medicine in chronic inflammatory disorders. Biochem. Pharmacol..

[41] Noer A., Boquest A.C., Collas P. (2007). Dynamics of adipogenic promoter DNA methylation during clonal culture of human adipose stem cells to senescence. BMC Cell Biol..

[42] Barres R., Yan J., Egan B., Treebak J.T., Rasmussen M., Fritz T., Caidahl K., Krook A., O’Gorman D.J., Zierath J.R. (2012). Acute exercise remodels promoter methylation in human skeletal muscle. Cell Metab..

[43] Barres R., Osler M.E., Yan J., Rune A., Fritz T., Caidahl K., Krook A., Zierath J.R. (2009). Non-CpG methylation of the PGC-1alpha promoter through DNMT3B controls mitochondrial density. Cell Metab..

[44] Milagro F.I., Campion J., Garcia-Diaz D.F., Goyenechea E., Paternain L., Martinez J.A. (2009). High fat diet-induced obesity modifies the methylation pattern of leptin promoter in rats. J. Physiol. Biochem..

[45] Bruggeman E.C., Garretson J.T., Wu R., Shi H., Xue B. (2018). Neuronal Dnmt1 Deficiency Attenuates Diet-Induced Obesity in Mice. Endocrinology.

[46] Cao Q., Wang X., Jia L., Mondal A.K., Diallo A., Hawkins G.A., Das S.K., Parks J.S., Yu L., Shi H. (2014). Inhibiting DNA Methylation by 5-Aza-2′-deoxycytidine ameliorates atherosclerosis through suppressing macrophage inflammation. Endocrinology.

[47] Yang X., Wang X., Liu D., Yu L., Xue B., Shi H. (2014). Epigenetic regulation of macrophage polarization by DNA methyltransferase 3b. Mol. Endocrinol..

[48] Yang Q., Liang X., Sun X., Zhang L., Fu X., Rogers C.J., Berim A., Zhang S., Wang S., Wang B. (2016). AMPK/alpha-Ketoglutarate Axis Dynamically Mediates DNA Demethylation in the Prdm16 Promoter and Brown Adipogenesis. Cell Metab..

[49] Michael L.F., Wu Z., Cheatham R.B., Puigserver P., Adelmant G., Lehman J.J., Kelly D.P., Spiegelman B.M. (2001). Restoration of insulin-sensitive glucose transporter (GLUT4) gene expression in muscle cells by the transcriptional coactivator PGC-1. Proc. Natl. Acad. Sci. USA.

[50] Shore A., Karamitri A., Kemp P., Speakman J.R., Lomax M.A. (2010). Role of Ucp1 enhancer methylation and chromatin remodelling in the control of Ucp1 expression in murine adipose tissue. Diabetologia.

[51] Gallagher D., Heymsfield S.B., Heo M., Jebb S.A., Murgatroyd P.R., Sakamoto Y. (2000). Healthy percentage body fat ranges: An approach for developing guidelines based on body mass index. Am. J. Clin. Nutr..

[52] Palmer B.F., Clegg D.J. (2015). The sexual dimorphism of obesity. Mol. Cell. Endocrinol..

[53] Schmidt S.L., Bessesen D.H., Stotz S., Peelor F.F., Miller B.F., Horton T.J. (2014). Adrenergic control of lipolysis in women compared with men. J. Appl. Physiol..

[54] Monteiro R., Teixeira D., Calhau C. (2014). Estrogen signaling in metabolic inflammation. Mediators Inflamm..

