# Supplementary material for: Brown Fat Dnmt3b Deficiency Ameliorates Obesity in Female Mice
Source: Life (Basel). 2021 Nov 30;11(12):1325. doi: 10.3390/life11121325 (PMC8703316; doi:10.3390/life11121325)
Supplement: Supplementary file 1 [file life-11-01325-s001.zip › life-1376635-supplementary.pdf]

life

MDPI

Supplementary Materials: Brown Fat Dnmt3b Deficiency Ameliorates Obesity in Female Mice

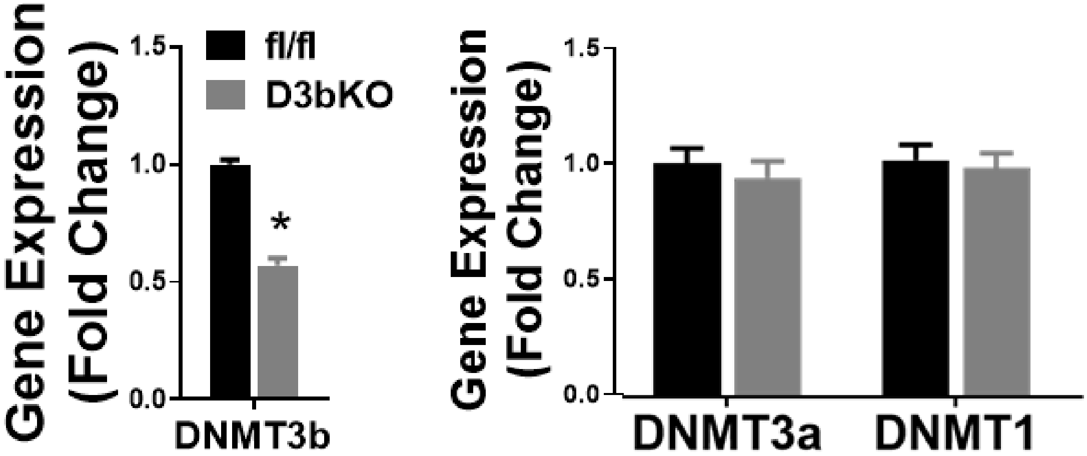

Figure S1. Dnmt3b, Dnmt3a and Dnmt1 mRNA levels in the interscapular BAT (iBAT) of female D3bKO and fl/fl mice (n = 4/group). All data are expressed as mean ± SEM; *$p < 0.05$ vs. fl/fl.

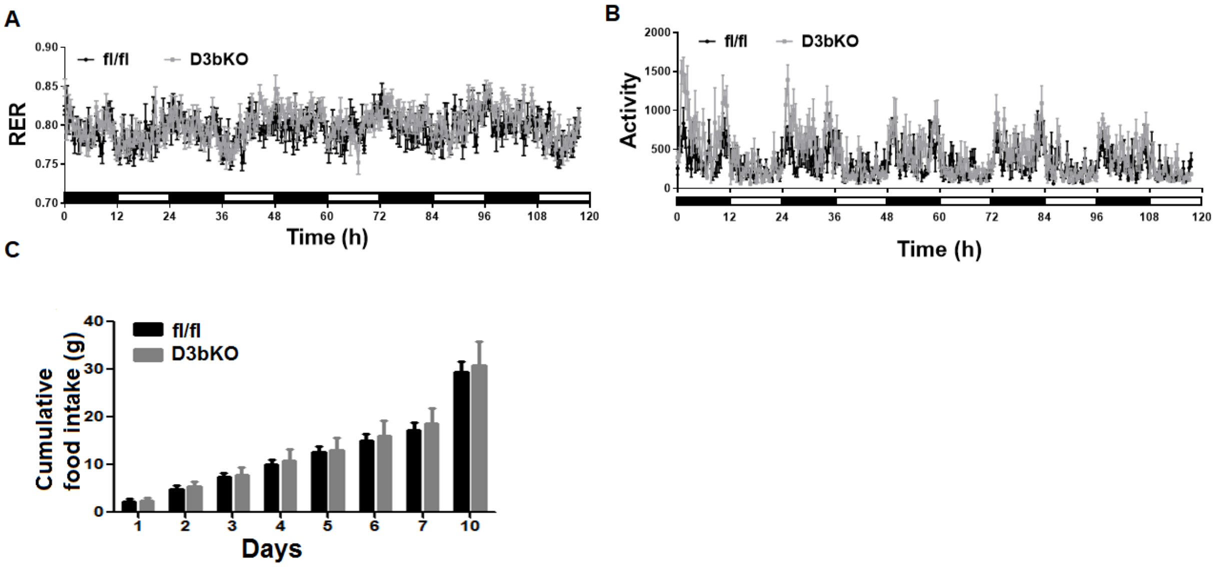

Figure S2. Metabolic characterization of female D3bKO and fl/fl control mice on HFD. 8-week old female D3bKO and their littermate control fl/fl mice were put on HFD for 20 weeks. Respiratory exchange ratio (RER) (**A**), locomotor activity (**B**), and food intake (**C**) were measured by TSE PhenoMaster metabolic cage systems in female D3bKO and fl/fl mice fed HFD. All data are expressed as mean ± SEM; n = 4/group; *$p < 0.05$ vs. fl/fl.

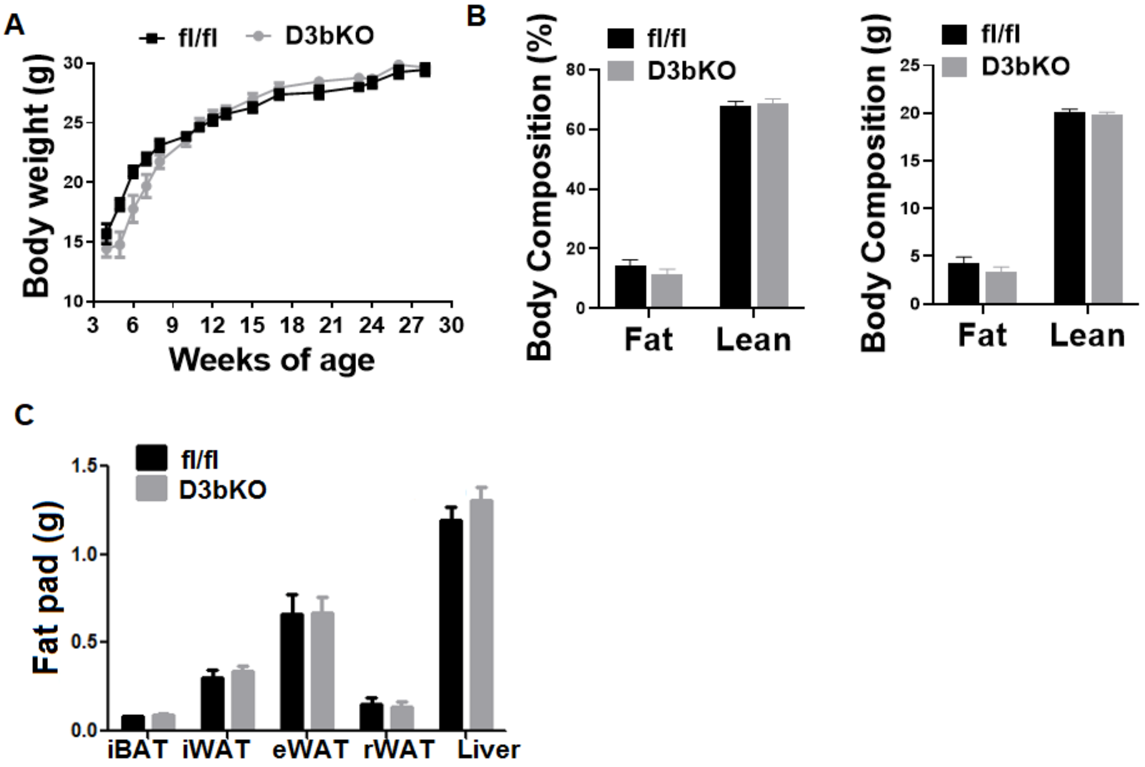

Figure S3. Dnmt3b deficiency in brown fat does not change body weight in male mice. 5-week old male D3bKO and their littermate control fl/fl mice were put on a regular chow diet for 25 weeks. (**A**) Body weight growth curve in male D3bKO and fl/fl mice. (**B**) Body composition measured by a Bruker NMR body composition analyzer in male D3bKO and fl/fl mice. (**C**) Organ weight of interscapular brown adipose tissue (iBAT), inguinal white adipose tissue (iWAT), gonadal WAT (gWAT), retroperitoneal WAT (rWAT), and liver in male D3bKO and fl/fl mice. All data are expressed as mean ± SEM; n = 8–11/group; **p* < 0.05 vs. fl/fl.

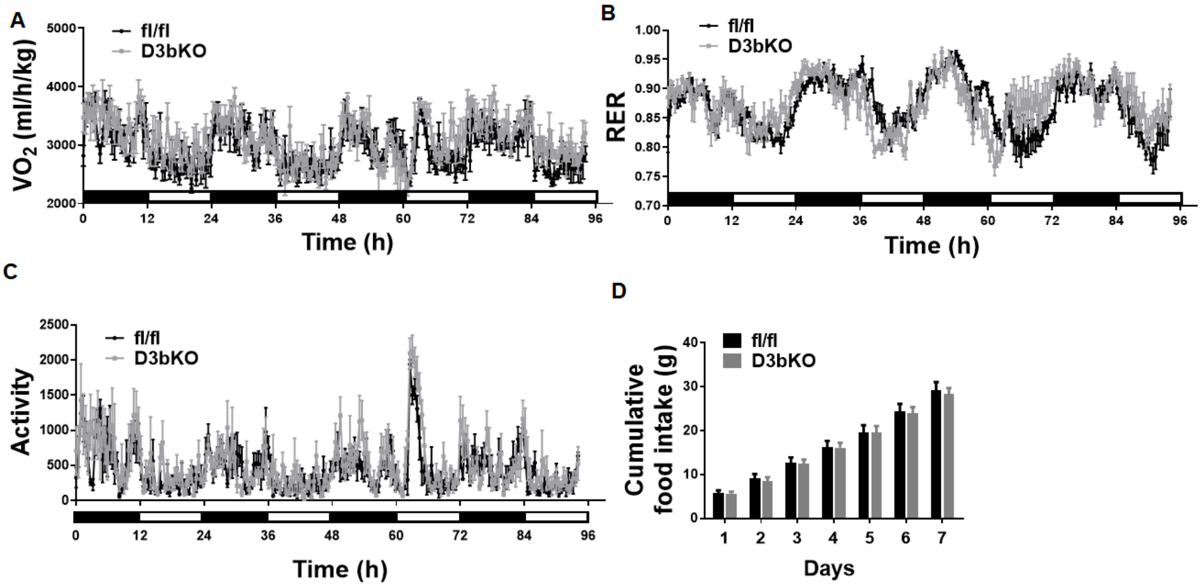

Figure S4. Dnmt3b deficiency in brown fat does not change energy expenditure in male mice. 29-week old male D3bKO and fl/fl mice fed a chow diet were put in TSE PhenoMaster metabolic cage system for the metabolic characterization. (**A**) Oxygen consumption. (**B**) Respiratory exchange ratio (RER). (**C**) Locomotor activity. (**D**) Cumulative food intake. All data are expressed as mean ± SEM; n = 4/group.

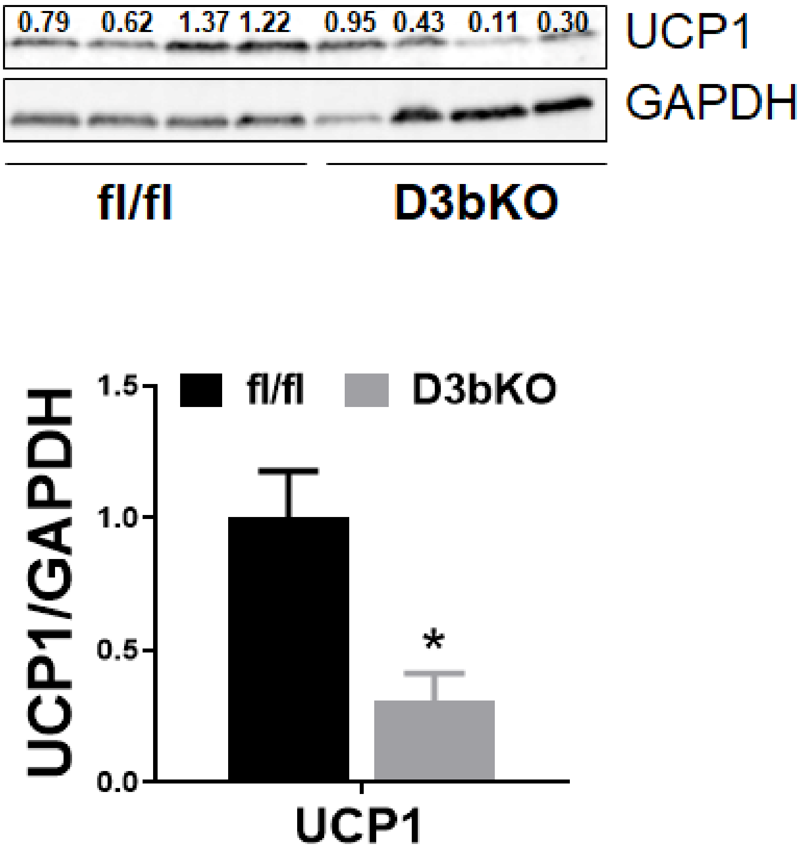

Figure S5. Dnmt3b deficiency reduces UCP1 protein levels in the iBAT of male mice. 5-week old male D3bKO and their littermate control fl/fl mice were put on a regular chow diet for 25 weeks. All data are expressed as mean ± SEM; n = 4/group; *$p < 0.05$ vs. fl/fl.

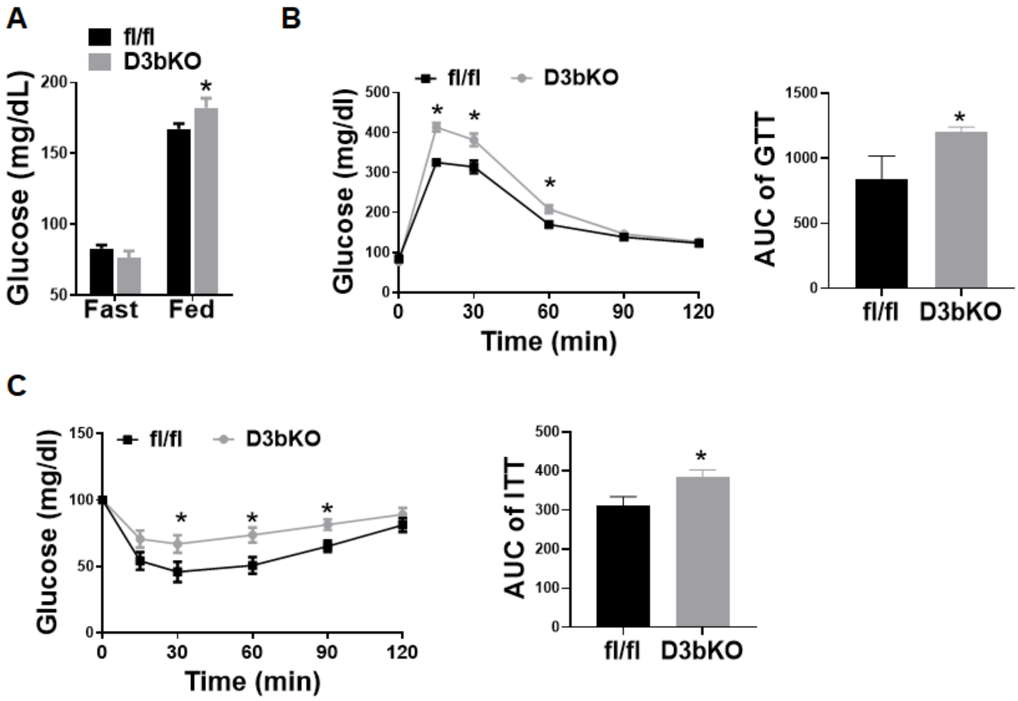

Figure S6. Dnmt3b deficiency in brown fat causes insulin resistance in male mice. 5-week old male D3bKO and their littermate control fl/fl mice were put on a regular chow diet for 25 weeks. (**A**) Fasting and fed glucose levels. (**B**) Glucose tolerance test (GTT) in 26-week male D3bKO and fl/fl mice. (**C**) Insulin tolerance test (ITT) in 28-week old male D3bKO and fl/fl mice. All data are expressed as mean ± SEM; n = 8–11/group.

Table S1. TaqMan primers/probes from Applied Biosystems.

Gene Symbol	Company	Catalog #
Dnmt3b	ABI	Mm01240111_g1
Acox1	ABI	Mm01246834_m1
Cidea	ABI	Mm00432554_m1
Dio2	ABI	Mm00515644_m1
Pgc1a	ABI	Mm01208835_m1
Pgc1b	ABI	Mm00504720_m1
Prdm16	ABI	Mm00712556_m1
Cpt1b	ABI	Mm00487191_g1
Otop1	ABI	Mm00554705_m1
Elovl3	ABI	Mm01194165_g1
